# The case of the missing appendix: a case report of appendiceal intussusception at the site of colonic mullerianosis

**DOI:** 10.1093/gastro/gov041

**Published:** 2015-09-16

**Authors:** Federico P Quirante, Lisandro M Montorfano, Federico Serrot, Mary E Billington, Giovanna Da Silva, Emanuele Lo Menzo, Samuel Szomstein, Raul J Rosenthal

**Affiliations:** * Department of Surgery, Cleveland Clinic Florida, Weston, FL, USA

**Keywords:** appendix, appendiceal intussusception, mullerianosis, endocervicosis

## Abstract

Right lower quadrant pain is a symptom with an exceptionally broad differential diagnosis. Intussusception of the appendix is a very uncommon condition with many manifestations. Additionally, the pathologic finding of ectopic presence of a mixture of at least two mullerian-derived tissue components is rare. This report presents the case of a 49-year-old woman who presented twice with acute right lower abdominal pain. Diagnosis of appendiceal inversion was made surgically. Pathologic examination of the specimen identified extensive endometriosis, endosalpingiosis and endocervicosis of the colon wall. Appendiceal intussusception and colonic mullerianosis, present together, are discussed, and recommendations for the diagnosis and treatment of appendiceal intussusception are discussed.

## Introduction

Intussusception of the appendix is a rare condition with an estimated incidence of 0.01% [1,2]. The incidence of appendicular intussusception is most frequent in adult women at an average age of 46 years [1]. This condition may present via many manifestations ranging from complete absence of symptoms to chronic symptoms (most common) to acute abdomen [1]. Inverted appendix is a rare diagnosis in the differential for abdominal pain syndromes, yet it is an important consideration because of the implications on patient management. An intussuscepted appendix may be simply reduced via contrast enema [3], removed via appendectomy, or removed with part of the cecum (appendectomy with removal of a “cecal cuff”) [1]. Misdiagnosis of the condition as a cecal tumor necessitates hemicolectomy, a much more invasive procedure with higher risks for a patient with a benign condition [4,5]. Diagnosis via radiographic imaging is difficult [1]. Enema, ultrasound and computed tomography (CT) are all potential diagnostic studies as long as the diagnosis is considered [3,6–8]. In the asymptomatic patient, diagnosis may be incidental during regular colonoscopy, when the inverted appendix may be mistaken for a polyp in the cecum base [9–14]. Polypectomy in this setting represents a high risk of perforation [11].

On the other hand, mullerianosis is a highly infrequent lesion diagnosed histologically by the presence of a mixture of at least two mullerian-derived tissue components [15]. Lesions included within Mullerian (paramesonephric) histology include endometriosis, endocervicosis and endosalpingiosis. Mullerianosis is rare and has been reported in the bladder and ureters [15,16] but never in the colon.

A low-population incidence, combined with poor diagnostic study options, in the setting of a disease characterized by a variety of clinical presentations make the diagnosis and appropriate treatment of appendix intussusception and mullerianosis difficult. Therefore, the purpose of this report is to present a case of appendiceal intussusception, analyze the diagnosis and treatment of this patient, examine the diagnostic options and discuss the appropriate management of the condition.

## Case report

This report presents the case of a 49-year-old Caucasian female with a past medical and surgical history significant for acute cholecystitis, for which she underwent a laparoscopic cholecystectomy in 2009, and symptomatic uterine fibroids, for which she underwent a laparoscopic supracervical hysterectomy and bilateral salpingectomy in 2012. At that time, evidence was noted of endometriosis at the left pelvic sidewall and posterior cul-de-sac. The patient also underwent colonoscopy in 2010, where a prominent appendiceal orifice was described. No additional medical conditions are reported.

**Figure 1.**
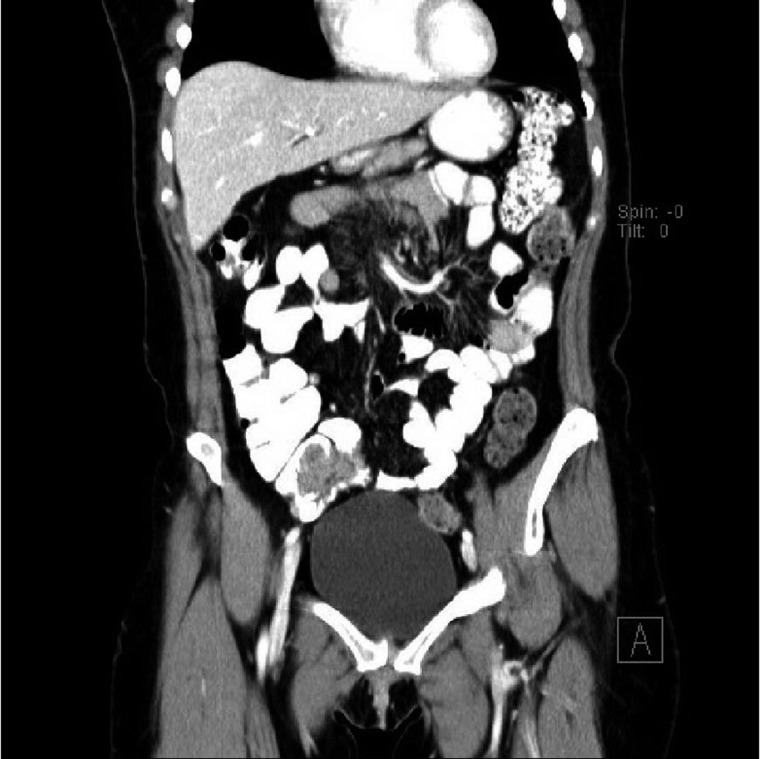
Contrast CT, coronal view of abdomen.

The patient presented to the emergency department complaining of a 5-day history of intermittent throbbing abdominal pain, initially periumbilical, but migrating to the right lower quadrant on initial presentation, which was associated with nausea and increased stool frequency. Physical exam was significant for moderate tenderness to palpation in the right lower quadrant and positive Rovsing sign. Laboratory studies were all within normal limits. Contrast CT identified lobulated tubular soft tissue and inflammation adjacent to the terminal ileum/ileocecal valve did not identify an appendix (Figure 1) and questioned whether the patient had previously undergone appendectomy. Without prior history of appendectomy, acute appendicitis was suggested as a highly likely diagnosis.

**Figure 2.**
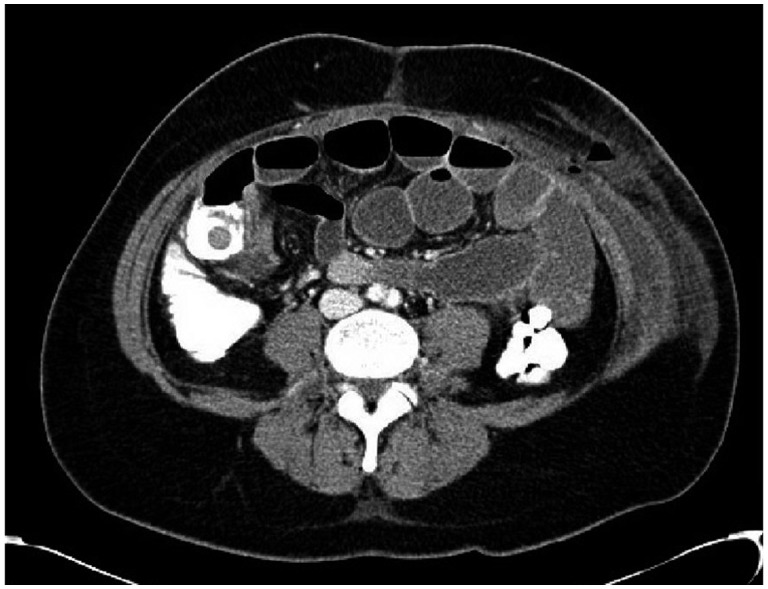
Contrast CT, transverse view of abdomen.

For this diagnosis, the patient was started on broad-spectrum antibiotics and underwent diagnostic laparoscopy. Mild inflammatory changes of the terminal ileum and adhesions involving the cecum, right ovary, and abdominal wall were observed without evidence of Crohn’s disease, but no appendix was identified despite full mobilization of the cecum and a thorough running of the small bowel. A biopsy of the inflamed areas was taken and revealed acute inflammatory changes surrounding the fallopian tubes as well as focuses of endometriosis. Intraoperative consultation with the colorectal surgery team was performed, and it was decided at that time to avoid further surgical interventions and continue course with intravenous antibiotics. Postoperatively, the patient began to tolerate a solid diet and was discharged on postoperative day 2, with plans for interval diagnostic colonoscopy. The patient returned to the emergency department within two hours of discharge, complaining of sharp right lower quadrant pain, nausea and vomiting. Repeat labs were significant for a white blood cell count of 13.23. Repeat CT of the abdomen and pelvis was consistent with small bowel obstruction focused at the level of the terminal ileum, likely secondary to an adjacent pericecal soft tissue mass (Figure 2). Differential diagnosis of the mass was inverted appendix versus neoplasm.

**Figure 3.**
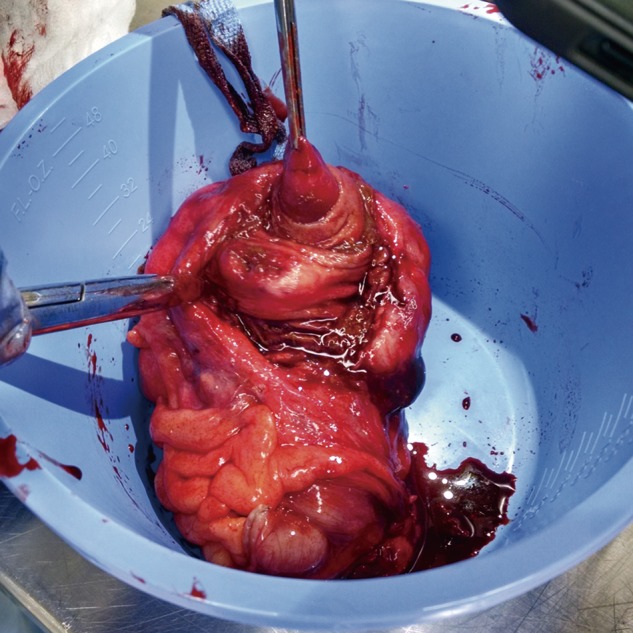
Resected right colon and inverted appendix.

**Figure 4.**
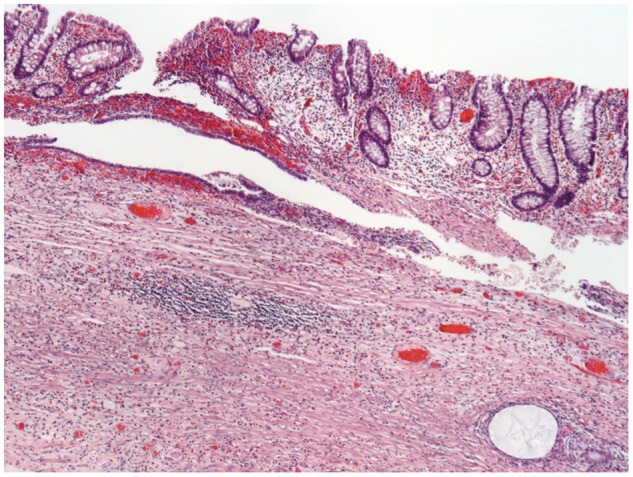
Pathology sample of colonic mullerianosis stained with hematoxylin and eosin.

Given the negative findings on prior diagnostic laparoscopy, a laparoscopic ileocecectomy with primary anastomosis was performed. Intraoperative inspection on the back table revealed a large inverted appendix. Gross pathologic examination identified an area of induration and fibrosis having a dark brown nodular appearance and located between the cecum and the terminal ileum. Within the bowel, an intussuscepted portion of appendix measuring 2.2 cm in length and up to 1.4 cm in diameter was identified; the appendix was not inflamed (Figure 3). Cut section through this projection showed fibrous cystic spaces filled with brown-tinged fluid. The remainder of the mucosal surface was tan with irregular, prominent, tightly spaced mucosal folds. Palpation revealed an area of induration that on cut section showed a thickened bowel wall measuring up to 0.7 cm in greatest dimension. Palpation of the underlying mesentery revealed palpable lymph nodes that were found to be negative for malignancy. Final pathologic diagnosis identified that the colonic wall was extensively involved by endometriosis, endosalpingiosis and endocervicosis (Figure 4).

The patient recovered after a short ileus and was successfully discharged home on a regular diet and having normal bowel function on postoperative day 7 from the second surgery.

## Discussion

Intussusception of the appendix is rare, affecting an estimated 0.01% of the population [1,2]. Additionally, not all cases of appendix intussusception are symptomatic. When symptomatic, the presentation is most frequently chronic [1]. Our report describes a case of appendiceal intussusception presenting with symptoms of relapsing acute abdomen. Imaging studies for our patient were consistent with the differential diagnosis of appendicitis versus malignancy.

Surgical management differs between these diagnoses. Obviously, the cases of clear appendicitis are treated surgically via appendectomy. Intussusception tends not to respond permanently to non-surgical management, and simple appendectomy may not be adequate treatment. Removal of a “cecal cuff” with the appendix is reasonable as this technique reduces the probability of recurrent intussusception of the appendix stump and creates a surgical margin for possible tumors at the base of the appendix [1]. Finally, malignancy is treated via right hemicolectomy. Due to these differences in operative management, it is imperative that the practicing surgeon be aware of this rare but benign and resectable diagnosis.

Inversion of the appendix represents a significant diagnostic challenge. Preoperative identification can be obtained by double-contrast barium enema (“coiled spring sign” with non-filling of the appendix), ultrasound (“doughnut sign”/”target lesion”), CT, endoscopy, and laparoscopy [1]. Our case suggests that this difficultly may arise in part due to non-inclusion of appendiceal intussusception in the differential diagnosis.

Based on the published cases in the literature, comparable proportions of patients treated with surgical therapy undergo appendectomy versus partial colectomy. Improving diagnostic algorithms and imaging may increase the rate of appropriate surgical treatment and decrease unnecessary resections. Therefore, this report reminds clinicians of this diagnosis and draws attention to the need for an accurate and precise diagnostic test to serve as the gold standard of diagnosis.

Mullerianosis is a rare lesion diagnosed histologically by the presence of a mixture of at least two Mullerian-derived tissue components [15]. Lesions included within Mullerian (paramesonephric) histology include endometriosis, endocervicosis and endosalpingiosis. These ectopic tissues, especially the endometrium, can cause scarring as well as produce pain-mediating factors (e.g. prostaglandins) that contribute to symptomatology. Mullerianosis is rare but has been reported in the bladder and ureters [15,16]; there are fewer than 20 reported cases of mullerianosis of the bladder [17]. A PubMed search of “mullerianosis colon” yields no results.

Endocervicosis is defined by the presence of benign ectopic endocervical-type glands. It is rarer than endometriosis and endosalpingiosis. It has been reported to involve the pelvic structures, but only one case of involvement of the small bowel has been reported [18]. One case of endocervicosis of the rectum has been reported [19] as has one case involving axillary lymph nodes [20]. To our knowledge, this is the first report of endocervicosis and mullerianosis of the large bowel.

Endocervicosis and endosalpingitis findings tend to be incidental, contrary to the more common endometriosis. The management of endocervicosis and endosalpingitis includes analgesics, hormonal therapy, surgical intervention or a combined approach [21]. The approach to management depends upon whether the goal is to ameliorate pain symptoms or treat infertility. However, in spite of extensive research, the optimal management remains unclear. The most common approach to surgical treatment is laparoscopic excision or ablation of ectopic implants [22].

Any commentary on inversion of the appendix is limited by the overall low incidence and diagnosis of the condition. Additionally, it is possible that not all cases are reported in the literature. Our case highlights the importance of reporting diagnosed events, such that practicing clinicians can diagnose and properly treat this patient population. Further studies on diagnosis of appendiceal intussusception may prove beneficial.

## Conclusion

This case reports a rare diagnosis of appendiceal intussusception, with pathology findings remarkable for the unique presence of endocervicosis and mullerianosis of the colon wall. This report adds data to the currently small body of literature for each finding. Additionally, diagnostic and treatment considerations for appendiceal intussusception were discussed, with the goal of educating the practicing clinician and reducing patient morbidity.

**Consent:** Written informed consent was obtained from the patient for publication of this case report and accompanying images. A copy of the written consent is available for review.

*Conflict of interest statement:* none declared.
